# Brucea javanica oil emulsion as an adjunct to chemoradiotherapy in cervical cancer: a systematic review and meta-analysis of adverse effects reduction and immune enhancement

**DOI:** 10.3389/or.2026.1880668

**Published:** 2026-08-24

**Authors:** Buwei Han, Mingge Liang, Ding Qi, Yangfan Qu, Jinzhe Liu, Wenxia Ai, Yue Yang, Wenlan Li

**Affiliations:** 1 School of Pharmacy, Harbin University of Commerce, Harbin, China; 2 Postdoctoral Scientific Research Workstation, Harbin University of Commerce, Harbin, China; 3 Graduate College, Heilongjiang University of Traditional Chinese Medicine, Harbin, China; 4 Gynecological Ward 2, Second Affiliated Hospital of Heilongjiang University of Chinese Medicine, Harbin, China; 5 School of Pharmacy, Guizhou University of Chinese Medicine, Guiyang, China

**Keywords:** adverse events, brucea javanica oil emulsion, cervical cancer, chemoradiotherapy, immune function, meta-analysis

## Abstract

**Background:**

Brucea javanica oil emulsion (BJOE) has been proposed as an adjuvant therapy to mitigate adverse reactions in cervical cancer patients receiving radiotherapy/chemotherapy, yet its efficacy remains understudied. This meta-analysis aimed to systematically evaluate the clinical benefits and safety of BJOE in this population.

**Methods:**

We searched PubMed, Embase, Cochrane Library, and six other databases for Randomized controlled trials (RCTs) up to February 2025. Primary outcomes included adverse reactions (rectal symptoms, nausea/vomiting, leukopenia, radiation cystitis) and immune function markers (CD3^+^, CD4^+^, CD8^+^). Pooled risk ratios (RR) and mean differences (MD) with 95% confidence intervals (CIs) were calculated using RevMan 5.4 and Stata 13.0.

**Results:**

Five RCTs (N = 372 patients) were included. BJOE supplementation significantly reduced the incidence of rectal symptoms (RR = 0.56, 95%CI: 0.45–0.68), nausea/vomiting (RR = 0.60, 95%CI: 0.43–0.85), leukopenia (RR = 0.52, 95%CI: 0.34–0.77), and radiation cystitis (RR = 0.46, 95%CI: 0.33–0.63). Immune function improved markedly (CD3+: MD = 4.42, 95%CI: 3.76–5.08; CD4+: MD = 2.50, 95%CI: 1.88–3.13; CD8+: MD = 3.14, 95%CI: 1.84–4.43).

**Conclusion:**

As an adjunctive rather than a direct antitumor agent, BJOE effectively reduces treatment-related adverse effects and enhances immune responses in patients with cervical cancer undergoing chemoradiotherapy, supporting its clinical utility. However, the current evidence grades predominantly fall into the low or very low categories, further large-scale trials are warranted to validate these findings.

## 1 Introduction

Cervical cancer (CC) is the fourth most common malignant tumor among women worldwide, exerting a negative impact on their quality of life and health. From the perspective of inducing factors, the vast majority of CC cases are associated with human papillomavirus (HPV) infection, and persistent HPV infection may progress to cervical intraepithelial neoplasia (CIN) or carcinoma *in situ* (1). Histopathologically, squamous cell carcinoma accounts for approximately 75% of cases, while adenocarcinoma constitutes around 20% (2). Clinically, surgical resection, radiotherapy, and systemic chemotherapy are the mainstay treatments for invasive cervical cancer. Most early-stage tumors are eligible for surgical resection, whereas radiotherapy alone or in combination with chemotherapy is the primary therapeutic approach for advanced-stage patients. However, cervical tumor lesions have a certain recurrence rate, which substantially compromises patients’ quality of life (3, 4). With advances in research, emerging therapeutic modalities such as immunotherapy (5), cell therapy (6), targeted therapy, and photodynamic therapy (7) have enriched the clinical treatment of CC. Combination regimens can alleviate the adverse effects of invasive treatments while enhancing clinical efficacy. In recent years, numerous studies on tumor prevention and treatment have highlighted the value of traditional Chinese medicine (TCM). Owing to its multi-targeted, multi-pathway actions, low drug resistance, cost-effectiveness, and definite therapeutic benefits, TCM exerts significant intervention effects on tumor-related diseases. Therefore, the innovation of multi-channel treatment, including TCM, is of great significance to improve the quality of life and prolong the survival time of patients.

Brucea javanica (BJ), a member of the *Brucea* SPP. family, exhibits pharmacological activities including detoxification, heat-clearing, anti-malarial, and anti-dysenteric effects. Studies have demonstrated that the extract of BJ has a strong affinity for tumor cells and can induce their apoptosis. Brucea javanica oil emulsion (BJOE) injection is an anticancer preparation for clinical application. Purified bruce javanica oil (BJO), soybean phospholipids, and glycerin serve as its excipients (8). BJOE is composed primarily of oleic acid, linoleic acid, and other fatty acids. Oleic acid and linoleic acid can prevent tumor cells from absorbing oxygen, inducing hypoxic cell death; furthermore, they can block tumor cell DNA synthesis. The surface activity of fatty acids can kill cancer cells, and their excessive accumulation can disrupt cancer cell membranes, leading to cell lysis. At the same time, the homogeneous formulation of the emulsion may enhance anti-tumor efficacy by facilitating targeted drug delivery (9).

The BJ extract has been most widely used in the treatment of digestive system tumors. Owing to its potent anti-cancer activity, it has recently been extended to the treatment of various malignancies, including lung cancer, breast cancer, CC, and leukemia. Additionally, it may attenuate the adverse effects of chemoradiotherapy and reverse multidrug resistance (10). An increasing percentage of clinical studies have been conducted on the use of BJOE in the treatment of CC; however, however, no comprehensive evaluation has been conducted on the efficacy of BJOE as an adjuvant therapy in reducing adverse reactions during CC chemoradiotherapy. This study aims to analyze the effectiveness of BJOE as an adjuvant therapy in lowering the risk of adverse events during CC chemoradiotherapy by leveraging clinical data. It is expected to enrich the clinical treatment of CC and provide evidence to support improvements in cancer patients’ survival outcomes.

## 2 Methods

This review was performed according to the Preferred Reporting Items for Systematic Reviews and Meta-Analyses (PRISMA) guidelines as well as the Cochrane Handbook for Systematic Reviews of Interventions. The protocol was registered on PROSPERO (Registration number: CRD42023450152).

### 2.1 Search strategy

We systematically searched the following databases from their inception to February 2025 for relevant randomized controlled trials (RCTs): PubMed, EMBASE, OVID, CINAHL, Cochrane Library, Clinical Trials, Chinese Clinical Trial Registry, and CNKI.

The search terms included:

#1: “Brucea javanica oil emulsion” (BJOE) OR “Javanica oil emulsion injection” OR “Brucea javanica oil”.
#2: “cervical cancer” OR “cervical neoplasms” OR “uterine cervical cancer”.
#3: “radiotherapy” OR “chemotherapy” OR “chemoradiotherapy”.
#4: #1 AND #2 AND #3.

Additionally, we manually screened the reference lists of included studies and those of relevant systematic reviews to identify potentially eligible trials.

### 2.2 Eligibility criteria and exclusion criteria

The PICOS criteria were used to screen and select the studies. Specific criteria are defined as follows:

Population: Patients with pathologically confirmed cervical cancer (any age, ethnicity, or histological subtype) who are receiving BJOE combined with chemoradiotherapy;

Intervention: BJOE combined with chemoradiotherapy (radiotherapy and/or chemotherapy);

Comparison: Chemoradiotherapy alone (radiotherapy and/or chemotherapy without BJOE);

Outcome: Adverse reactions and total effective rate. ①Adverse reactions: rectal symptoms, nausea and vomiting, leukopenia, skin injury, elevated serum transaminase, radiation cystitis, bone marrow suppression, and immune function evaluation (CD3^+^, CD4^+^, CD8^+^). ②Overall response rate (ORR): Evaluated according to the World Health Organization efficacy evaluation criteria (11), which classifies responses into complete response (CR), partial response (PR), stable disease (SD), and progressive disease (PD). Total response rate (%) = (CR + PR)/total number of cases ×100%;

#### 2.2.1 Study design: RCTs

Studies were excluded if they met any of the following criteria:

① Non-RCT designs (e.g., reviews, case reports, conference abstracts, experimental animal studies); ② Duplicate publications or overlapping datasets; ③ Unavailable full-text or incomplete outcome data; ④ Interventions involving non-pharmacological therapies (e.g., surgery, acupuncture) in either group.

### 2.3 Literature selection and data extraction

In this study, two researchers (BW-H, MG-L) independently conducted literature screening and data extraction, respectively. Duplicate records were removed by EndNote software, followed by preliminary screening of titles and abstracts by two independent reviewers. Subsequently, the full texts were reviewed against the inclusion and exclusion criteria to determine the final eligibility of each study. The following data were obtained from the eligible articles: basic information of the literature, baseline characteristics of patients, intervention measures in both the experimental and control groups, treatment duration, and outcome indicators. The finally collected data were cross-checked, and any discrepancies were resolved by a third researcher.

### 2.4 Risk of bias assessment

Two authors (BW-H, MG-L) utilized the Cochrane risk of bias tool to assess the methodological quality of RCTs. Each bias was categorized as either “High risk of bias”, “Low risk of bias”, or “Unclear risk of bias” (12). Any discrepancies between the two reviewers were resolved through discussion or consultation with a third author (WL-L) until a unanimous consensus was reached.

### 2.5 Statistical analysis

The data were analyzed using Revman 5.4 and Stata 13.0 software. The Relative Risk (RR) with associated 95% confidence intervals (CIs) was calculated for dichotomous variables to represent the difference between the groups. For continuous data, the results were shown as a mean difference (MD) and 95% CI of the variations between the intervention group and the control group before and after the medication. Chi-square was used to analyze the heterogeneity of the included studies, and *I* ^*2*^ was used to evaluate it. For meta-analysis, when *P* > 0.1 and *I* ^*2*^ < 50%, Fixed effects model was used; in addition, a random effects model was utilized. After the results are obtained, *P* ≤ 0.05 indicates significant results. Funnel plots were drawn for publication bias analyses and sensitivity analyses were performed using Stata 13.0 to assess the stability of the results.

### 2.6 Quality assessment

According to the GRADE approach, the quality of evidence is categorized into four grades: high (indicating we are very confident that the true effect lies close to the estimated value), moderate (reflecting moderate confidence that the true effect is proximate to the estimated value), low (denoting limited confidence in the effect estimate), and very low (signifying minimal confidence that the observed effect approximates the estimated value) (13). By default, each outcome is initially rated as high quality and subsequently downgraded to one of the aforementioned categories based on an assessment of five predefined domains. Two independent reviewers appraised the eligibility criteria and conducted a systematic evaluation of the literature. For those with a disagreement, the third party (Wenlan Li) discussed the decision to reach an agreement.

## 3 Results

### 3.1 Literature retrieval and screening results

**FIGURE 1:**
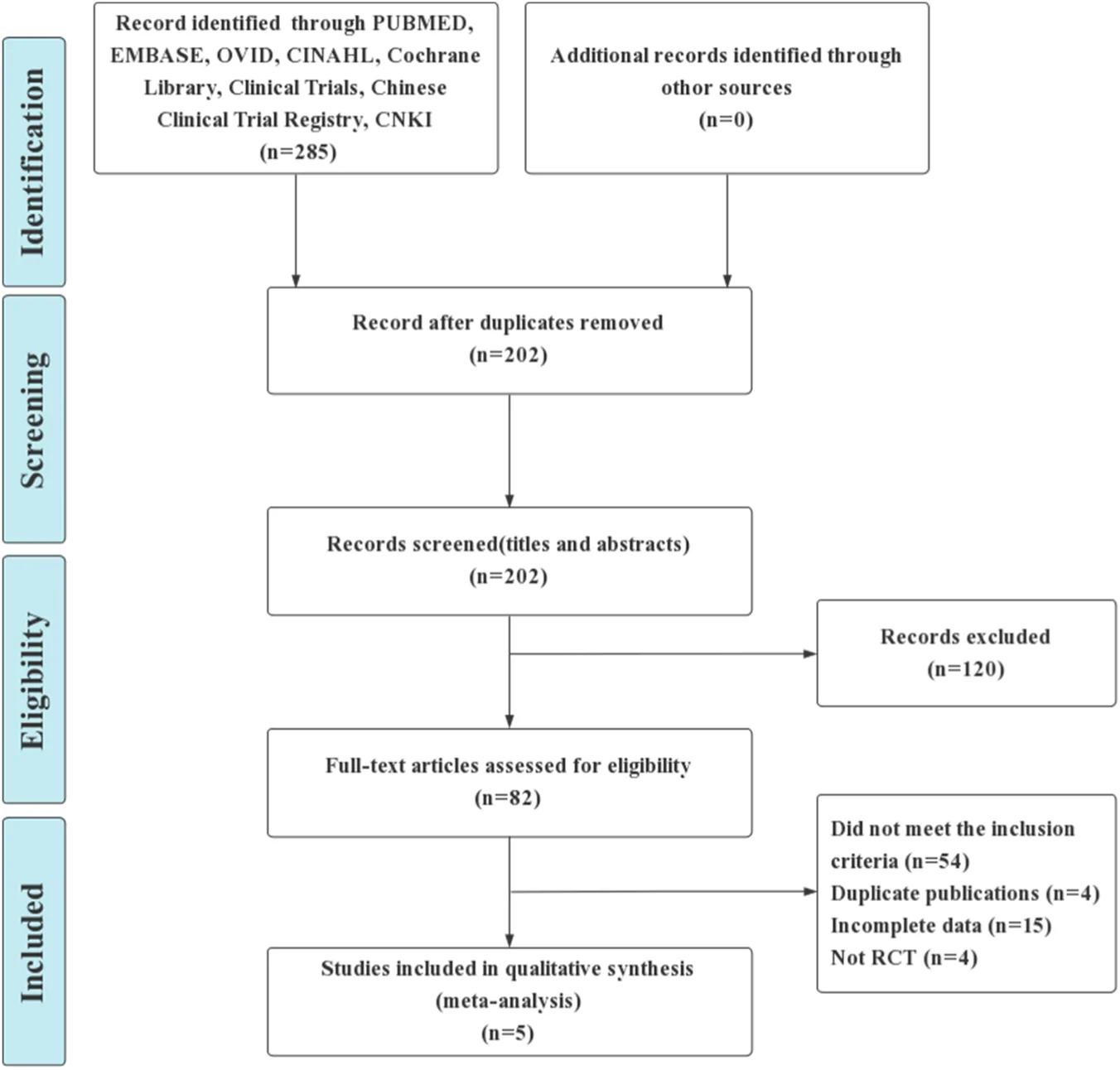
PRISMA flow diagram for literature screening process and results.

285 conceivably relevant articles were imported into NoteExpress after searches across database. Following a preliminary review, 202 papers were still available. After carefully reading each article’s title and abstract, 120 pieces of research that didn't fit with the requirements were removed. Subsequently, the remaining 82 articles were further screened while taking into factors such as accurate clinical data, article quality, and duplicate researchers. As a result, only 5 articles fulfilled the criteria. The process and results of the literature screening are illustrated in Figure 1.

### 3.2 Characteristics of the studies

372 subjects, comprising 183 in the observation group and 189 in the control group, were included among the 5 RCTs that were all Chinese literary works for this study. The basic information of the included studies is shown in Table 1.

**TABLE 1:** Overall characteristics of the included studies.

First author/year	Sample size		Age (year)		Intervention		Course(d)	Outcomes
	T	C	T	C	T	C		
Peng FH/2022 (12)	48	48	51.2 ± 3.4	49.9 ± 3.5	BJOE +RT + CT	RT + CT	21 × 4	①②③ ④⑤⑨
Yang CM/2016 (13)	42	44	45	45	BJOE +RT + CT	RT + CT	40–60	①②③④ ⑤⑥⑨
SHANG YP/2016 (14)	31	31	39.0 ± 7.5	39.0 ± 7.5	BJOE +RT + CT	RT + CT	30+	①②③④⑤⑥⑦⑧⑨
Deng SH/2014 (15)	34	34	43	43	BJOE +RT	RT	21	⑥⑦⑨
WU HH/2013 (16)	28	32	53	55	BJOE +RT	RT	28	①⑥⑧⑨

T: observation group, C: control group, RT: radiotherapy, CT: chemotherapy. Outcome indicators: ① rectal reaction; ② Nausea and vomiting; ③ leukopenia; ④ skin injury; ⑤ Elevated serum transaminase; ⑥ Immune function evaluation; ⑦ Myelosuppression; ⑧ Radiation cystitis; ⑨ Total clinical effective rate.

### 3.3 Risk of bias assessment

**FIGURE 2:**
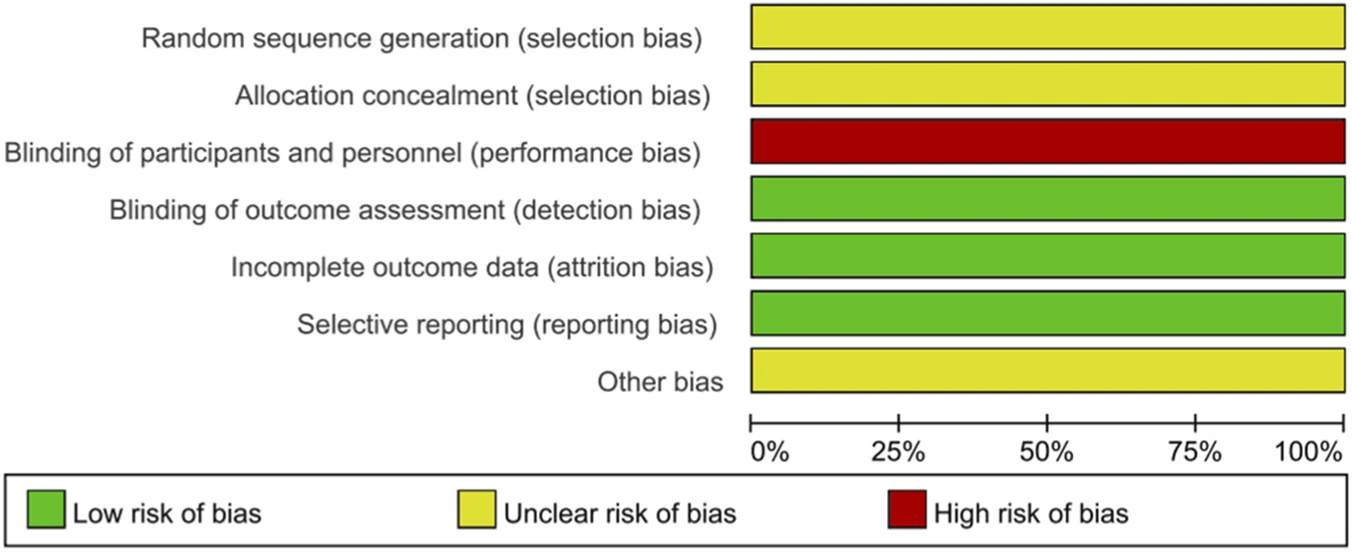
Risk bias evaluation diagram of the included literature.

The baselines in the five included studies were consistent, and the interventions were parallel. The methods used to generate the random sequences were not described in any of the five randomized controlled investigations, which rendered it impossible to assess the grade risk, all of the risks were unknown. There was insufficient information to assess the grade risk, which was all unknown risks, in any of the five trials, none of which stated the allocation process. The five studies did not mention blindness. It is unlikely that any of them will be blinded, though, given the experimental group in this study used different intervention measures than the control group did, and that there was no placebo control. Even if blindness is set, blindness may be broken, and the outcome may be affected by the absence of blindness. Because the test results of CC patients are objective experimental test data, and the outcome is not subject to the subjective influence of the result evaluator, the blind method in the evaluation result measurement process is determined to be low risk. There were no missing data or non-selective reporting of study results in all literature, so 5 studies were judged to be of low risk. Because the five included studies did not have enough information to determine whether there was high or low bias, other biases were judged to be unknown risks. The overall picture of the quality assessment is shown in Figure 2.

### 3.4 Outcomes

#### 3.4.1 Rectal symptoms

**FIGURE 3:**
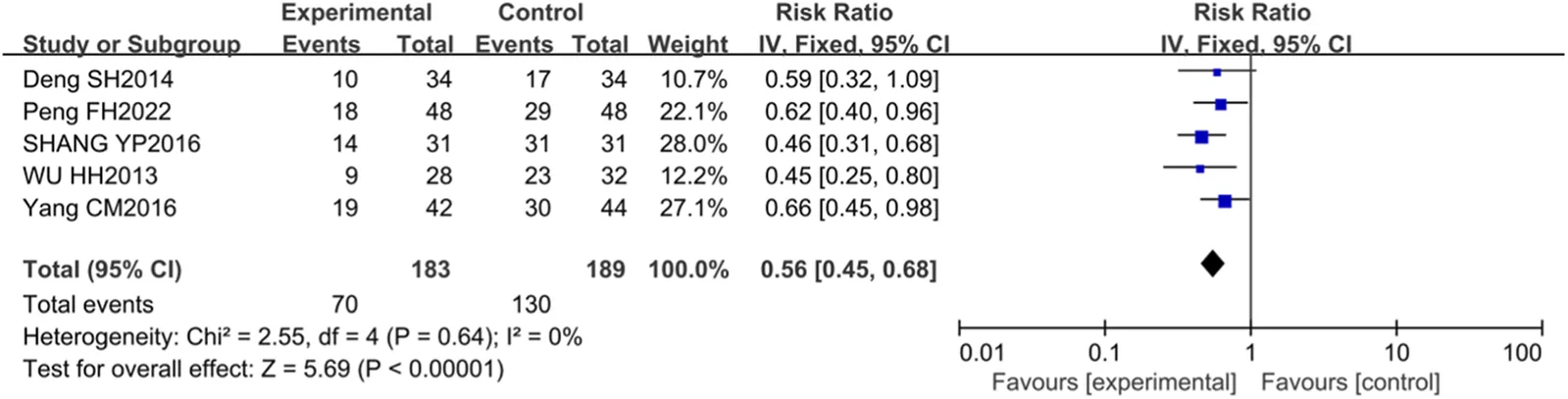
Forest plot of rectal symptoms.

A total of five studies (14–18) reported rectal symptoms. Since the heterogeneity results showed *P* = 0.64 and *I* ^*2*^ = 0%, a fixed-effect model was selected for analysis in this study. The results showed that the probability of rectal symptoms was significantly reduced in the treatment group compared with the control group (*RR* = 0.56, *95%CI*:0.45–0.68, *P* < 0.00001), as shown in Figure 3. Consistent results were reported in the sensitivity analysis,in File 2. Funnel plots based on the five included studies showed approximate symmetry, indicating limited influence of publication bias, in File 3.

#### 3.4.2 Nausea and vomiting

**FIGURE 4:**
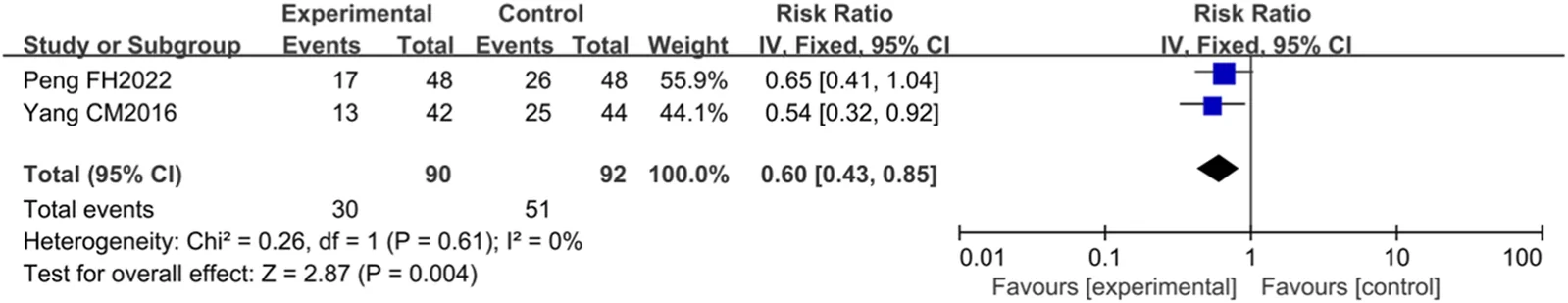
Forest plot of nausea and vomiting.

A total of 2 studies (14, 15) reported nausea and vomiting. Since the heterogeneity results showed *P* = 0.61 and *I* ^*2*^ = 0%, a fixed-effect model was selected for analysis in this study. The results showed that compared with the control group, the probability of nausea and vomiting could be reduced in the treatment group to some extent. Further this was statistically proven to be significant. (*RR* = 0.60, *95%CI*: 0.43–0.85, *P* = 0.004), as shown in Figure 4. Only two studies in this dataset were unsuitable for sensitivity analysis, and the stability of the meta-analysis results remains uncertain. Funnel plots based on the two included studies showed approximate symmetry, indicating limited influence of publication bias, in File 3.

#### 3.4.3 Leukopenia

**FIGURE 5:**
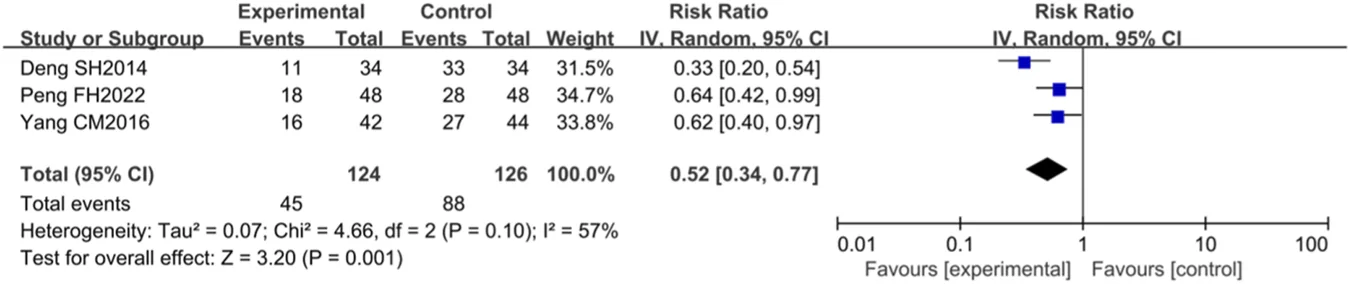
Forest plot of leukopenia.

A total of three studies (14, 15, 17)reported leukopenia. Since the heterogeneity results showed *P* = 0.10 and *I* ^*2*^ = 57%, a random effects model was selected for analysis in this study. The results showed that compared with the control group, the probability of leukopenia could be reduced in the treatment group to some extent. However, it was statistically significant (*RR* = 0.52, *95%CI*:0.34–0.77, *P* = 0.001), as shown in Figure 5. Sensitivity analysis showed the same results after excluding each study one by one, in File 2. Leave-one-out sensitivity analysis demonstrated that the overall pooled estimate was robust, with no substantial variation when any single study was excluded,in File 2. Funnel plots based on the three included studies showed approximate symmetry, indicating limited influence of publication bias, in File 3.

#### 3.4.4 Skin injury

**FIGURE 6:**
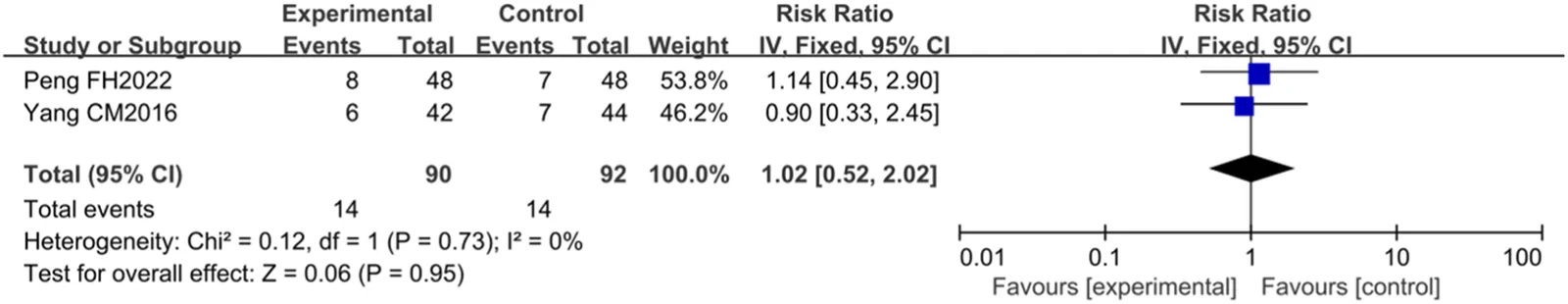
Forest plot of skin injury.

A total of 2 studies (14, 15) reported skin injury. Since the heterogeneity results showed P = 0.73 and I2 = 0%, a fixed-effect model was selected for analysis in this study. The results showed that compared with the control group, the treatment group could not reduce the probability of skin injury to a certain extent. (*RR* = 1.02, *95%CI:*0.52–2.02, *P* = 0.95), as shown in Figure 6. Only two studies in this dataset were unsuitable for sensitivity analysis, and the stability of the meta-analysis results remains uncertain. Funnel plots based on the two included studies showed approximate symmetry, indicating limited influence of publication bias, in File 3.

#### 3.4.5 Elevated serum aminotransferase

**FIGURE 7:**
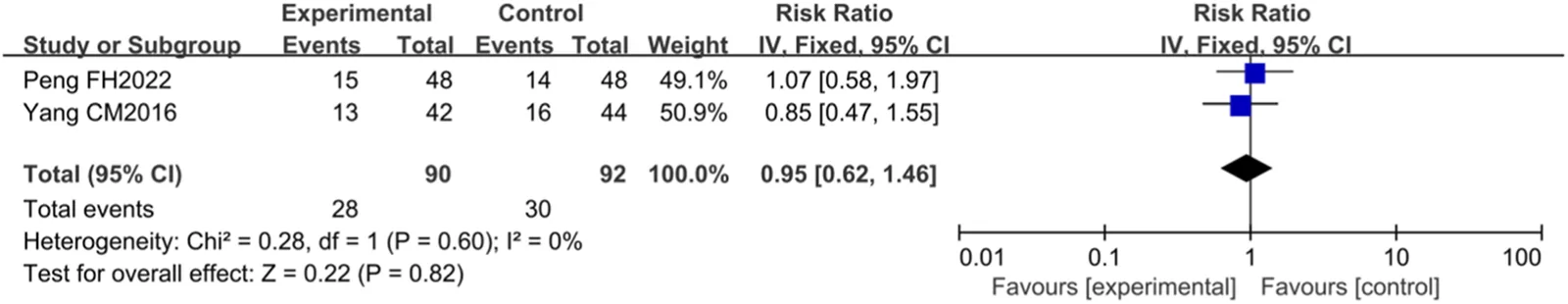
Forest plot of elevated serum aminotransferase.

A total of two studies (14, 15) reported elevated serum transaminase. Since the heterogeneity results showed *P* = 0.60 and *I* ^*2*^ = 0%, a fixed-effect model was selected for analysis in this study. The results showed that compared with the control group, the probability of serum transaminase increase could not be reduced in the treatment group to some extent. (*RR* = 0.95, *95%CI*:0.62–1.46, *P* = 0.82), as shown in Figure 7. Only two studies in this dataset were unsuitable for sensitivity analysis, and the stability of the meta-analysis results remains uncertain. Funnel plots based on the two included studies showed approximate symmetry, indicating limited influence of publication bias, in File 3.

#### 3.4.6 Radiation cystitis

**FIGURE 8:**
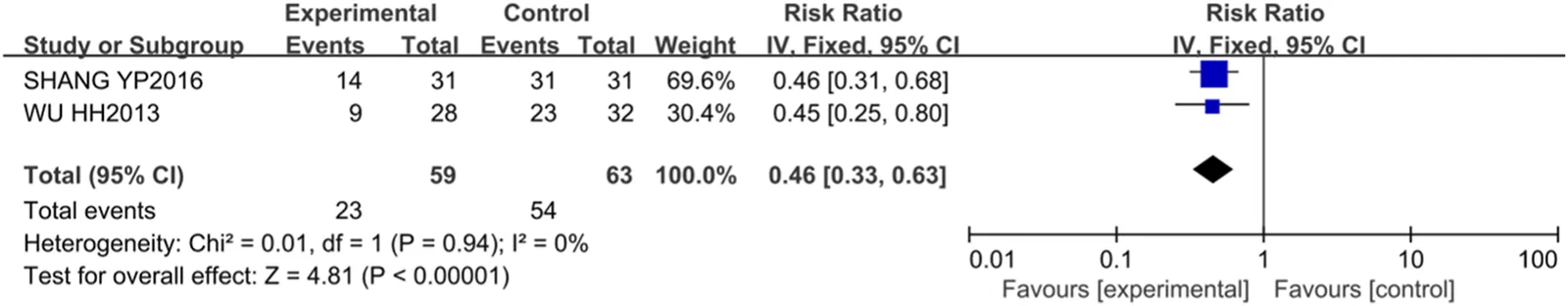
Forest plot of radiation cystitis.

A total of 2 studies (16, 18) reported radiation cystitis. Since the heterogeneity results showed *P* = 0.94 and *I* ^*2*^ = 0%, a fixed-effect model was selected for analysis in this study. The results showed that compared with the control group, the treatment group could significantly reduce the probability of radiation cystitis to some extent. (*RR* = 0.46, *95%CI*:0.33–0.63, *P* < 0.00001), as shown in Figure 8. Only two studies in this dataset were unsuitable for sensitivity analysis, and the stability of the meta-analysis results remains uncertain. Funnel plots based on the two included studies showed approximate symmetry, indicating limited influence of publication bias, in File 3.

#### 3.4.7 Myelosuppression

**FIGURE 9:**
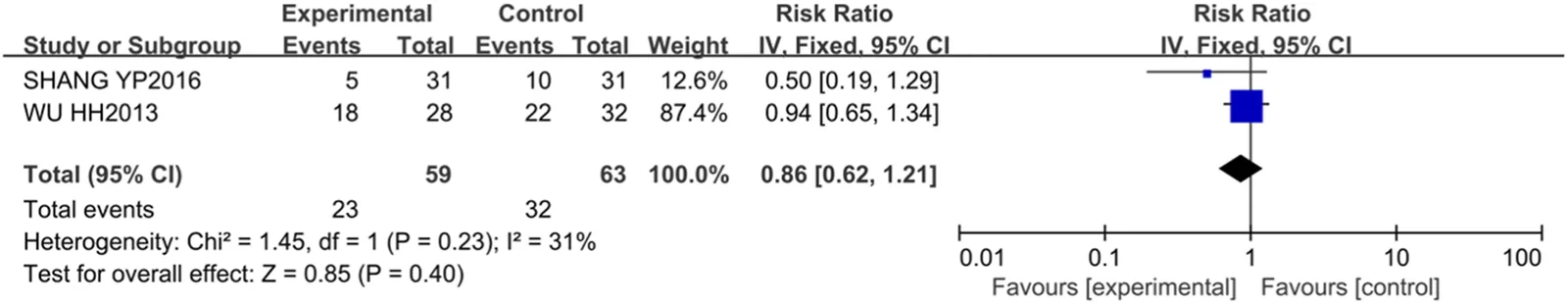
Forest plot of myelosuppression.

A total of 2 studies (16, 18) reported myelosuppression. Since the heterogeneity results showed *P* = 0.40 and *I* ^*2*^ = 31%, a fixed-effect model was selected for analysis in this study. The results showed that compared with the control group, the probability of myelosuppression could be reduced in the treatment group to some extent. This was statistically significant (*RR* = 0.86, *95%CI*:0.62–1.21, *P* = 0.40), as shown in Figure 9. Only two studies in this dataset were unsuitable for sensitivity analysis, and the stability of the meta-analysis results remains uncertain. Funnel plots based on the two included studies showed approximate symmetry, indicating limited influence of publication bias, in File 3.

#### 3.4.8 Immune function evaluation

**FIGURE 10:**
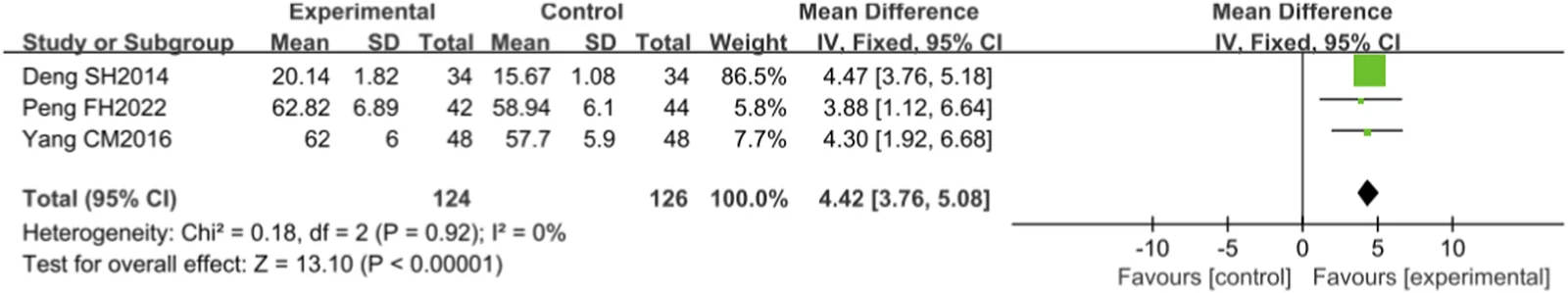
Forest plot of CD3^+^.

**FIGURE 11:**
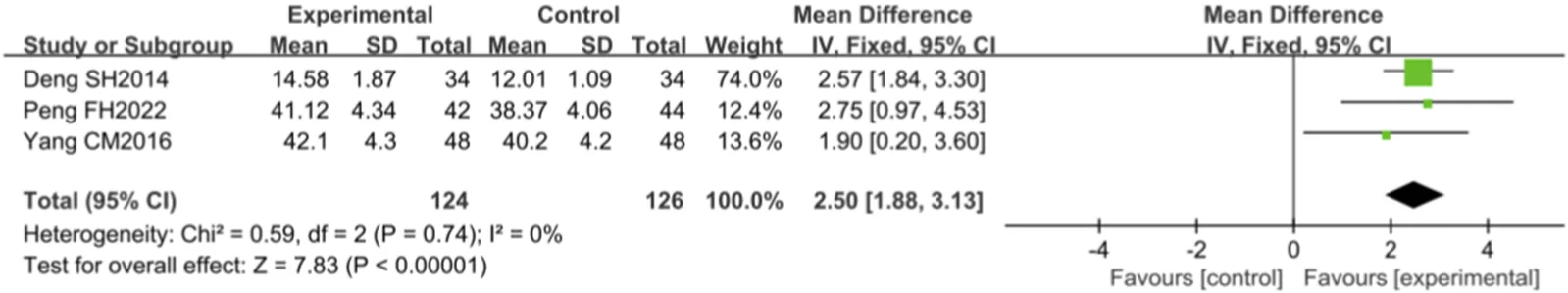
Forest plot of CD4^+^.

**FIGURE 12:**
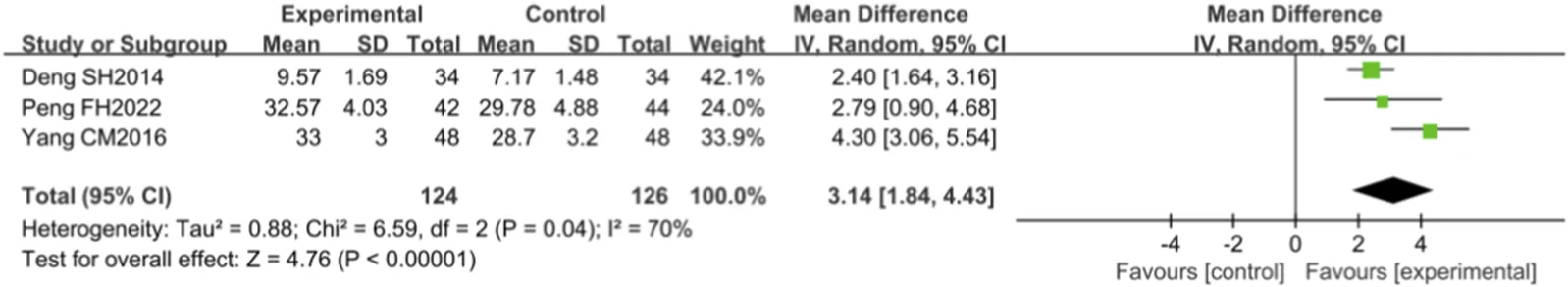
Forest plot of CD8^+^.

A total of three studies (14, 15, 17) reported detection of immune function. CD3^+^ (*MD* = 4.42, *95%CI*:3.76–5.08, *P* < 0.00001); CD4^+^ (*MD* = 2.50, *95%CI*:1.88–3.13, *P* < 0.00001); CD8^+^ (*MD* = 3.14, *95%CI*:1.84–4.43, *P* < 0.00001). The results showed that compared with the control group, the treatment group could improve the immune function to a certain extent, as shown in Figures 10–12. Sensitivity analysis showed the same results after excluding each study one by one, in File 2. Funnel plots based on the three included studies showed approximate symmetry, indicating limited influence of publication bias, in File 3.

#### 3.4.9 Clinical total effective rate

**FIGURE 13:**
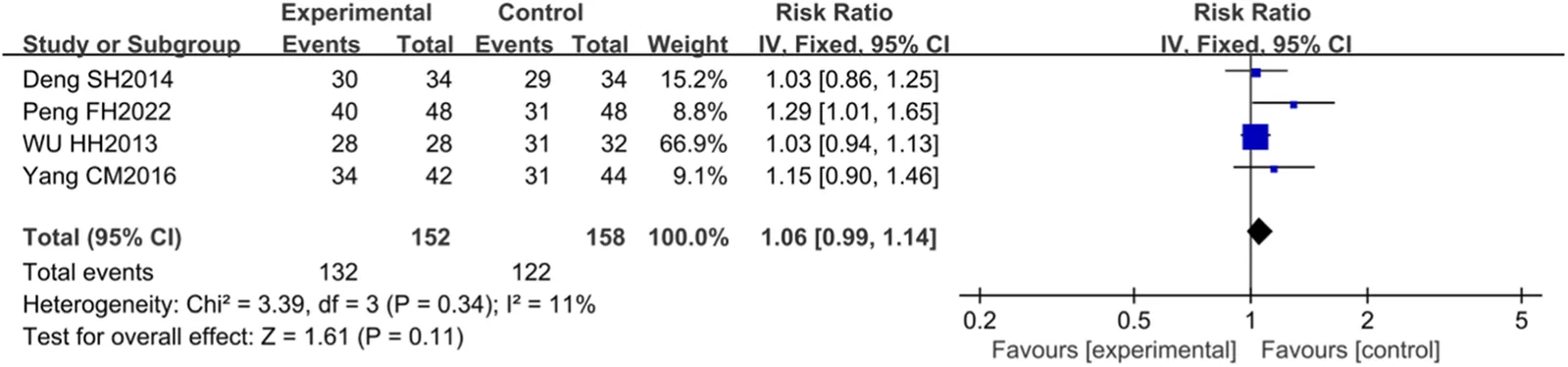
Forest plot of total effective rate.

A total of four studies (14, 15, 17, 18) reported overall clinical response rates. Since the heterogeneity results showed *P* = 0.34 and *I* ^*2*^ = 11%, a fixed-effect model was selected for analysis in this study. The results showed that compared with radiotherapy/chemotherapy alone, the total response rate of BJOE combined therapy for CC was not significantly improved, and the difference between the two groups was not statistically significant (*RR* = 1.06, *95%CI*:0.99–1.14, *P* = 0.11), as shown in Figure 13. Sensitivity analysis showed the same results after excluding each study one by one, in File 2. Funnel plots based on the four included studies showed approximate symmetry, indicating limited influence of publication bias, in File 3.

#### 3.4.10 Grade

There were 9 outcomes that were measured included in the meta-analysis. Among these outcomes, high quality of evidence was found in none (0.0%), moderate in (11%), low in 6 (67%), and very low in 2 (22%). Regarding the five downgrading elements, the most common items were risk of bias (n = 9, 100%), imprecision (n = 9, 100%), inconsistency (n = 2, 22%), indirectness (n = 0, 0%) and publication bias (n = 0, 0%) (Table 2).

**TABLE 2:** GRADE for quality of evidence profile.

Outcomes (number of studies)	Risk of bias	Inconsistency	Indirectness	Imprecision	Publication bias	Quality of evidence
①(5)	Serious^a^	Not serious	Not serious	Serious^c^	Undetected	Moderate
②(2)	Serious^a^	Not serious	Not serious	Serious^c^	Undetected	Low
③(3)	Serious^a^	Serious^b^	Not serious	Serious^c^	Undetected	Very low
④(2)	Serious^a^	Not serious	Not serious	Serious^c^	Undetected	Low
⑤(2)	Serious^a^	Not serious	Not serious	Serious^c^	Undetected	Low
⑥(2)	Serious^a^	Not serious	Not serious	Serious^c^	Undetected	Low
⑦(2)	Serious^a^	Not serious	Not serious	Serious^c^	Undetected	Low
⑧(3)	Serious^a^	Serious^b^	Not serious	Serious^c^	Undetected	Very low
⑨(4)	Serious^a^	Not serious	Not serious	Serious^c^	Undetected	Low

^a^(Unclear random sequence generation, allocation concealment blinding not done in all studies); ^b^(The overlap degree of different research confidence intervals is poor, and *I* ^*2*^ > 50%); ^c^(Inadequate sample size and the wide 95% (CI)).

Abbreviations: ① rectal reaction; ② Nausea and vomiting; ③ leukopenia; ④ skin injury; ⑤ Elevated serum transaminase; ⑥ Immune function evaluation; ⑦ Myelosuppression; ⑧ Radiation cystitis; ⑨ Total clinical effective rate.

## 4 Discussion

The cancer of the cervix is a multi-step process, and it usually takes many years from HPV infection to cancer. It is necessary to develop new interventions to control cancer since, notwithstanding the fact that the creation of preventative vaccines may somewhat lower the incidence of CC, the incidence of CC can be markedly reduced for at least 20 years (19). According to the Results of SEER (Surveillance, Epidemiology and End Results) project in the United States, 36% of CC patients are under 45 years old, and some of them hope to preserve fertility (20), so radiotherapy/chemotherapy has become the primary choice for many women. A randomized trial showed that there was no difference in survival between surgery and radiotherapy for CC patients with stage IB1 to stage IIA (21). The overall clinical response rate can be increased by combining chemotherapy with extracorporeal radiation treatment and brachytherapy. However, patients may experience varying degrees of acute gastrointestinal reactions, hematological toxicity, and drug resistance. The most important prognostic factors for advanced CC are stage, response to radical chemoradiotherapy and complete response after chemotherapy (22). Therefore, it is of great significance to seek effective means to improve tumor sensitivity to chemoradiotherapy and reduce adverse reactions associated with it.

In the domain of cancer supportive care, a number of traditional Chinese medicine injections-including Shenqi Fuzheng Injection and Kang’ai Injection-have been studied as adjunctive therapies for radiotherapy and chemotherapy, with effects primarily focused on regulating systemic immune function or improving quality of life (23). Compared with conventional Chinese herbal adjuvant therapies, BJOE shows better efficacy in relieving chemotherapy-induced myelosuppression. Unlike antiemetics or granulocyte colony-stimulating factor (G-CSF), which only target specific adverse events such as gastrointestinal reactions or neutropenia, BJOE provides more comprehensive hematopoietic protection. Similar bone marrow-protective effects have also been recorded in meta-analyses focused on esophageal and hepatocellular carcinomas (14, 18). Therefore, BJOE holds a unique position in the supportive care for cervical cancer, mainly through alleviating myelotoxic side effects caused by chemotherapy regimens.

BJOE has become one of the most widely studied Chinese medicine preparations for the treatment of cancer (24). Numerous investigations have demonstrated that BJOE can exert a synergistic anti-tumor effect by enhancing tumor response, improving quality of life, and lowering the frequency of adverse events during radiotherapy/chemotherapy (25–27). However, BJOE features a multi-directional dynamic unstable dispersion system and an improper temperature may result in separation. BJOE binds to malignant cells with special affinity. The anti-tumor mechanism of BJOE may include: (1) inhibiting DNA synthesis of cancer cells; (2) damaging the biofilm structure of tumor cells; (3) increasing the permeability of tumor cell membrane to anticancer drugs (9); (4) inducing apoptosis of tumor cells (26); (5) decreasing the expression of cancer stem cell markers; (6) enhancing cellular immunity (28); and (7) protecting bone marrow stem cells. Meanwhile, the nutritional value of soy phospholipid oil in BJOE injection is quite high, and it has a protective impact on the mucosa of the digestive tract. Plenty of preparations that are more effective, stable, and efficient have been developed with the help of science and technology, including gastric retention floating beads, self-microemulsion, nano-emulsion, liposome, nano-structured lipid carrier and sponge (29–31). The optimization of preparations can better enhance the inhibitory effect of BJOE on tumor cell growth (28, 32). Because T lymphocyte subsets can reflect changes in host immune regulatory function, we found that the addition of BJOE could not only decrease the likelihood of some adverse reactions, but also protect and improve the immune function of patients. In tumor immunology research, peripheral blood and tumor-infiltrating lymphocyte subpopulations have been extensively investigated as prognostic biomarkers. Accumulating evidence indicates that high-density infiltration of CD4^+^ and CD8^+^ T cells is significantly positively correlated with better disease-specific survival. Furthermore, derivative indicators such as the CD4^+^/CD8^+^ ratio have also been demonstrated to be closely associated with immunotherapy response and overall survival (33, 34). This was manifested in the increased levels of T lymphocyte subsets (CD3^+^, CD4^+^, CD8^+^). The fall of its value indicates that the cellular immune function of the patient is decreased, whereas the reverse indicates that the cellular immune function is enhanced (35). However, current evidence is insufficient to determine whether the modest increments observed in this study are sufficient to cross such clinically relevant thresholds or translate into detectable clinical benefits. Therefore, these findings should be interpreted as a positive signal that the treatment has triggered an immune response, while the direct clinical benefits remain to be confirmed through long-term follow-up and functional validation. Moderate heterogeneity (I^2^ = 57%) existed in the pooled analysis of leukopenia incidence. Although we adopted the random-effects model to synthesize the data, we failed to quantitatively explore the sources of heterogeneity. The core constraint is that only a small number of trials reported this indicator, which fails to meet the minimum sample size requirement for conducting subgroup analysis and meta-regression.

Several critical limitations weaken the certainty of our pooled results, consistent with the GRADE assessment. First, all included RCTs were conducted in a single country, limiting population generalizability. Most trials enrolled small sample sizes; our sensitivity analysis confirmed that removing small-cohort studies slightly attenuated the pooled therapeutic benefit, suggesting small trials may overestimate the efficacy of BJOE (36). Second, primary studies carried a high overall risk of bias. Many trials lacked clear random sequence generation, allocation concealment and double-blinding, with incomplete reporting of follow-up and missing intention-to-treat analysis. Sensitivity analysis excluding high-bias trials visibly reduced the observed adjuvant advantage, indicating flawed trial design positively skews treatment effects. Third, asymmetric funnel plots implied potential publication bias. Small trials with neutral or negative outcomes are likely unpublished, leading to an overoptimistic pooled effect size and downgrading GRADE evidence certainty. Besides, trials only reported short-term tumor response and acute toxicities without long-term survival data, and the dosage of BJOE varied greatly across studies, resulting in substantial heterogeneity. Taken together, these drawbacks restrict clinical extrapolation. We have softened all conclusions in the manuscript to avoid overinterpreting the herbal adjuvant benefit, and large multinational rigorous RCTs are needed to verify our findings.

## 5 Conclusion

Based on existing evidence, BJOE addition therapy reduces the risk of adverse reactions caused by CC radiotherapy/chemotherapy, such as rectal symptoms, nausea and vomiting, leukopenia, radiation cystitis, and decreased immune function. It has recommended value for clinical use. It should be noted that BJOE is positioned as a supportive care agent for toxicity reduction rather than an anti-tumor drug. However, the current evidence grades predominantly fall into the low or very low categories. The conclusions of the meta-analysis require further validation through larger-scale, well-designed, and rigorously conducted randomized double-blind controlled trials.

## Data Availability

The original contributions presented in the study are included in the article/supplementary material, further inquiries can be directed to the corresponding author.

## References

[1] Ye J Zheng L He Y Qi X . Human papillomavirus associated cervical lesion: pathogenesis and therapeutic interventions. MedComm (2020) 4(5):e368. 10.1002/mco2.368 PMC10501338 37719443

[2] Cohen CM Wentzensen N Castle PE Schiffman M Zuna R Arend RC Racial and ethnic disparities in cervical cancer incidence, survival, and mortality by histologic subtype. J Clin Oncol (2023) 41(5):1059–68. 10.1200/JCO.22.01424 36455190 PMC9928618

[3] Zhong L Li K Song L Yin R . The effect of consolidation chemotherapy after concurrent chemoradiation on the prognosis of locally advanced cervical cancer: a systematic review and meta-analysis. J Obstet Gynaecol (2022) 42(5):830–7. 10.1080/01443615.2021.2012437 35148230

[4] Van Den Akker MJE Horeweg N Beltman JJ Creutzberg CL Nout RA . Efficacy and toxicity of postoperative external beam radiotherapy or chemoradiation for early-stage cervical cancer. Int J Gynecol Cancer (2020) 30(12):1878–86. 10.1136/ijgc-2019-001131 32591371

[5] Sherer MV Kotha NV Williamson C Mayadev J . Advances in immunotherapy for cervical cancer: recent developments and future directions. Int J Gynecol Cancer (2022) 32(3):281–7. 10.1136/ijgc-2021-002492 35256414

[6] Zhou X Lian H Li H Fan M Xu W Jin Y . Nanotechnology in cervical cancer immunotherapy: therapeutic vaccines and adoptive cell therapy. Front Pharmacol (2022) 16(13):1065793. 10.3389/fphar.2022.1065793 PMC9802678 36588709

[7] Razlog R Kruger CA Abrahamse H . Enhancement of conventional and photodynamic therapy for treatment of cervical cancer with cannabidiol. Integr Cancer Ther (2022) 21(21):15347354221092706. 10.1177/15347354221092706 35481367 PMC9087227

[8] Wang Y Chen B Xiao M Wang X Peng Y . Brucea javanica oil emulsion promotes autophagy in ovarian cancer cells through the miR-8485/LAMTOR3/mTOR/ATG13 signaling axis. Front Pharmacol (2022) 25(13):935155. 10.3389/fphar.2022.935155 35959437 PMC9358144

[9] Li Q Chen X Lin W Guo X Ma Y . Application of a novel multicomponent nanoemulsion to tumor therapy based on the theory of unification of drugs and excipients. Pharm Dev Technol (2023) 28(3-4):351–62. 10.1080/10837450.2023.2196330 36971746

[10] Zhang J Xu HX Dou YX Huang QH Xian YF Lin ZX . Major constituents from Brucea javanica and their pharmacological actions. Front Pharmacol (2022) 18(13):853119. 10.3389/fphar.2022.853119 PMC8971814 35370639

[11] Aykan NF Ozatli T . Objective response rate assessment in oncology: current situation and future expectations. World J Clin Oncol (2020) 11(2):53–73. 10.5306/wjco.v11.i2.53 32133275 PMC7046919

[12] Flemyng E Moore TH Boutron I Higgins JP Hróbjartsson A Nejstgaard CH Using risk of bias 2 to assess results from randomised controlled trials: guidance from cochrane. BMJ Evid Based Med (2023) 28(4):260–6. 10.1136/bmjebm-2022-112102 36693715

[13] Brozek JL Canelo-Aybar C Akl EA Bowen JM Bucher J Chiu WA GRADE guidelines 30: the GRADE approach to assessing the certainty of modeled evidence-An overview in the context of health decision-making. J Clin Epidemiol (2021) 129(1):138–50. 10.1016/j.jclinepi.2020.09.018 32980429 PMC8514123

[14] Peng FH Li L . Analysis of effects of Brucea javanica oil emulsion injection on treatment of cervical cancer and improvement of adverse reactions of chemoradiotherapy. J Clin Pharmacother (2022) 20(4):26–31. (in Chinese).

[15] Yang CM . Clinical Observation of Simultaneous Chemoradiotherapy Combined with Brucea javanica Oil Softgel in the Treatment of Advanced Cervical Cancer [D]. Liaoning University of Traditional Chinese Medicine (2016). (in Chinese).

[16] Shang YP Liu BL Ma JH Teng YL Chen C Wang ZM Nursing analysis of Brucea javanica oil emulsion in alleviating radiation and chemotherapy reaction of cervical cancer. Chongqing Med (2016) 45(2):285–7. (in Chinese).

[17] Deng SH Chen P . Clinical observation of Brucea javanica oil emulsion combined with intensity modulated radiotherapy and intracavitary retrofitting in treatment of cervical cancer. Mod Oncol Med (2014) 22(8):1928–30. (in Chinese).

[18] Wu HH Liang H Li YX . Clinical observation of intensity modulated radiotherapy combined with brucea javanica oil emulsion injection in the treatment of 28 cases of early cervical cancer. Hebei Traditional Chin Med (2013) 35(2):236–8. (in Chinese).

[19] Lei J Ploner A Elfstrom KM Wang J Roth A Fang F HPV vaccination and the risk of invasive cervical cance. N Engl J Med (2020) 383(14):1340–8. 10.1056/NEJMoa1917338 32997908

[20] Zaccarini F Sanson C Maulard A Schérier S Leary A Pautier P Cervical cancer and fertility-sparing treatment. J Clin Med (2021) 10(21):4825. 10.3390/jcm10214825 34768345 PMC8585101

[21] Chen CS Huang EY . Comparison of oncologic outcomes between radical hysterectomy and primary concurrent chemoradiotherapy in women with bulky IB and IIA cervical cancer under risk stratification. Cancers (Basel) (2023) 15(11):3034. 10.3390/cancers15113034 37296994 PMC10252013

[22] Chopra S Goda JS Mittal P Mulani J Pant S Pai V Concurrent chemoradiation and brachytherapy alone or in combination with nelfinavir in locally advanced cervical cancer (NELCER): study protocol for a phase III trial. BMJ Open (2022) 12(4):e055765. 10.1136/bmjopen-2021-055765 PMC8987785 35387819

[23] Yao YL Li HG Chen ZW Xu WJ Zhou L Zhou ZY Clinical study of jianpi wenshen ruanjian jiedu formula combined with Kang'ai injection and low-dose chemotherapy in the treatment of elderly patients with advanced non-small cell lung cancer of spleen-kidney deficiency pattern. Shanghai J Traditional Chin Med (2020) 54(3):67–71. (in Chinese).

[24] Zhang D Wang K Zheng J Wu J Duan X Ni M Comparative efficacy and safety of Chinese herbal injections combined with transcatheter hepatic arterial chemoembolization in treatment of liver cancer: a bayesian network Meta-analysis. J Tradit Chin Med (2020) 40(2):167–87. 32242383

[25] Luo D Hou D Wen T Feng M Zhang H . Efficacy and safety of Brucea javanica oil emulsion for liver cancer: a protocol for systematic review and meta-analysis. Medicine (Baltimore) (2020) 99(47):e23197. 10.1097/MD.0000000000023197 33217831 PMC7676520

[26] Ye L Zhao JF Wang YM Chen WH Qian S Zhou ZG Brucea javanica oil emulsion suppresses tumor growth in human cervical cancer cells through inhibition of the E6 oncogene and induction of apoptosis. Transl Cancer Res (2020) 9(2):918–29. 10.21037/tcr.2019.12.62 35117437 PMC8797272

[27] Shi R Wu Z Wang H Zhang J Zhang F Stalin A Investigation on the efficiency of Brucea javanica oil emulsion injection with chemotherapy for treating malignant pleural effusion: a meta-analysis of randomized controlled trials. Front Pharmacol (2022) 16(13):998218. 10.3389/fphar.2022.998218 36188623 PMC9524220

[28] Li K Xiao K Zhu S Wang Y Wang W . Chinese herbal medicine for primary liver cancer therapy: perspectives and challenges. Front Pharmacol (2022) 5(13):889799. 10.3389/fphar.2022.889799 35600861 PMC9117702

[29] Bi D Zhao L Yu R Li H Guo Y Wang X Surface modification of doxorubicin-loaded nanoparticles based on polydopamine with pH-sensitive property for tumor targeting therapy. Drug Deliv (2018) 25(1):564–75. 10.1080/10717544.2018.1440447 29457518 PMC6058689

[30] Huang T Wang Y Shen Y Ao H Guo Y Han M Preparation of high drug-loading celastrol nanosuspensions and their anti-breast cancer activities *in vitro* and *in vivo* . Sci Rep (2020) 10(1):8851. 10.1038/s41598-020-65773-9 32483248 PMC7264310

[31] Hong J Liu Y Xiao Y Yang X Su W Zhang M High drug payload curcumin nanosuspensions stabilized by mPEG-DSPE and SPC: *in vitro* and *in vivo* evaluation. Drug Deliv (2017) 24(1):109–20. 10.1080/10717544.2016.1233589 28155567 PMC8253124

[32] Yoon BK Lim ZY Jeon WY Cho NJ Kim JH Jackman JA . Medicinal activities and nanomedicine delivery strategies for Brucea javanica oil and its molecular components. Molecules (2020) 25(22):5414. 10.3390/molecules25225414 33228061 PMC7699344

[33] Caramelo O Almeida V Fidalgo A Cipriano A Almeida-Santos T . Prognostic relevance of specific TIL (CD4+, CD8+, and FOXP3 + T-cell infiltrates) in triple-negative breast cancer: short- and long-term outcomes. Breast Cancer (2026) 33(2):386–95. 10.1007/s12282-025-01819-y 41498919 PMC12960379

[34] Wang C Liu F Jia Y Yan Y Tan X Yuan X Peripheral blood IFN-gamma-producing T-cell subsets and soluble IL-2 receptor as independent prognostic biomarkers in NSCLC treated with immune checkpoint inhibitor-based therapy. Discov Oncol (2026) 6(8):68. 10.1007/s12672-026-05343-z PMC13473051 42258128

[35] Wang YY Zhou N Liu HS Gong XL Zhu R Li XY Circulating activated lymphocyte subsets as potential blood biomarkers of cancer progression. Cancer Med (2020) 9(14):5086–94. 10.1002/cam4.3150 32459060 PMC7367640

[36] Lin L Shi L Chu H Murad MH . The magnitude of small-study effects in the cochrane database of systematic reviews: an empirical study of nearly 30 000 meta-analyses. BMJ Evid Based Med (2020) 25(1):27–32. 10.1136/bmjebm-2019-111191 31273125 PMC6942244

